# Prevalence and risk factors for obstructive pulmonary dysfunction caused by silica dust exposure: a multicenter cross-sectional study

**DOI:** 10.1186/s12890-024-03106-6

**Published:** 2024-06-25

**Authors:** Li Xin, Tang Mei An, Li Ying, Dai Wei Rong, Huang Lei

**Affiliations:** Hunan Prevention and Treatment Institute for Occupational Diseases, Changsha, China

## Abstract

**Objective:**

To understand the prevalence rate of obstructive pulmonary dysfunction in workers exposed to silica dust and analyze its risk factors, so as to provide reference for the formulation of diagnostic criteria for chronic obstructive pulmonary disease caused by occupational dust.

**Methods:**

Data collection and structured questionnaire were used to collect the data of 2064 workers exposed to silica dust who underwent health examination in Hunan Occupational Disease Prevention and Control Hospital and Yuanling Second People’s Hospital from January 1, 2021 to June 30, 2022. The prevalence rate of obstructive pulmonary ventilation dysfunction was analyzed and the risk factors were analyzed.

**Results:**

The prevalence rate of obstructive pulmonary ventilation dysfunction (FEV1/FVC < 70%) was 2.3% in 2064 silica dust exposed workers. The prevalence of restrictive pulmonary ventilation dysfunction (FVC/Pre < 80%) was 8.1%. The prevalence of obstructive pulmonary ventilation dysfunction in the high level exposure group was higher than that in the low level exposure group, 8.2 vs0.9% (*P* < 0.05). The rate of obstructive pulmonary ventilation dysfunction in female group was higher than that in male group (5.3% vs. 1.7%, *p* = 0.00). Workers with obstructive pulmonary dysfunction were older and worked longer than workers without obstructive pulmonary dysfunction, but there was no statistical difference. Multivariate regression analysis showed that high exposure level was a risk factor for obstructive pulmonary ventilation dysfunction in silica dust exposed workers (*P* < 0.05). Females were the risk factors for obstructive pulmonary ventilation dysfunction (*P* < 0.05).

**Conclusion:**

Silica dust exposure can cause obstructive pulmonary ventilation dysfunction and lead to chronic obstructive pulmonary disease. High level of exposure is a risk factor for obstructive pulmonary ventilation dysfunction. Women exposed to dust are more prone to obstructive pulmonary ventilation dysfunction than men. Early diagnosis of chronic obstructive pulmonary disease caused by silica dust and timely intervention measures are very important to delay the decline of lung function and protect the health of workers.

## Background

Chronic Obstructive Pulmonary Disease (Chronic Obstructive Pulmonary diseases, COPD) is a heterogeneous lung condition, with Chronic respiratory symptoms (dyspnea, cough, sputum), is due to airway (bronchitis, Bronchiolitis) and/or abnormalities in the alveoli (emphysema) that result in persistent (often progressive) airflow obstruction is a common, preventable and treatable chronic airway disease [1]. The 2019 Burden of Disease Study (GBD) states that COPD is the third leading cause of death globally [2]. Due to its high prevalence, disability and mortality, COPD has become a worldwide public health problem [3–5].

The risk factors of COPD include smoking, occupational dust, air pollutants, and genetic, developmental, and social factors [1]. A number of systematic assessments have concluded that there is indeed a causal relationship between occupational exposure and COPD [6], and the ILO Occupational Diseases catalogue includes inhalation of coal dust from work activities, dust from quarries (mines), wood dust, and other pollutants. COPD is caused by dust from grain and agricultural work, dust from animal stables (sheds), dust from textiles and paper dust. The EU list of occupational diseases includes COPD caused by coal dust and silica dust. Taiwan of China has made reference guidelines for the determination of COPD in underground coal miners with reference to European Union standards, and the list of occupational diseases in South Korea includes COPD caused by coal dust and cadmium smoke. In 2011, China issued the national occupational health standard of the People’s Republic of China, “Diagnosis of Chronic obstructive pulmonary disease caused by occupational irritating chemicals” (GBZ/T237-2011) standard, because of the lack of large sample epidemiological studies and evidence, did not include occupational dust.

This study intends to evaluate information from annual physical examination from multiple centers ( Hunan Occupational Disease Prevention Hospital, Yuanling County Second People ' s Hospital ), to investigate the prevalence rate of obstructive pulmonary ventilation dysfunction (FEV1/FVC < 70%) among workers exposed to silica dust at work in Minmetals Nonferrous Metals Group ( affiliated to Yaogangxian Tungsten Mine ) and Hunan Gold Group ( affiliated to 4 gold mines ) and analyze its risk factors .

## Methods

Prospective study method was used to collect data.The Ethics committee of Hunan Prevention and Treatment Institute for Occupational Diseases approved the study and waived the written informed consent.

2.1 Research subjects: The Hunan Provincial Occupational Disease Prevention and Control Hospital and the Second People’s Hospital of Yuanling County are designated physical examination hospitals for dust workers in Hunan Province. The study focuses on the on duty silicon dust exposed workers who were voluntarily arranged by the unit to undergo physical examinations at these two hospitals from January 1, 2021 to June 30, 2022, including those from Wukuang Colorful Group (a subsidiary of Yaogangxian Tungsten Mine) and Hunan Gold Group (a subsidiary of four gold mines).

Exclusion criteria: Imaging showed pneumonia, tuberculosis and other lung diseases; Pulmonary function data is incomplete or missing.

A total of 2277 cases were included according to the inclusion criteria, 213 cases were excluded according to the exclusion criteria, and the final health monitoring data of 2064 workers exposed to silica dust on the job were included in the analysis.

2.2 In this study, we collected workplace dust concentration, occupational history (including dust exposure type, exposure start time, exposure end time, job type), age, sex, smoking history, chest X-ray results, and pulmonary function test results (FEV1/FVC, FVC/Pre). The workplace dust concentration is obtained from the unit’s most recent annual monitoring report.

The diagnosis of silicosis was made by 3 occupational disease specialists (including 2 occupational disease clinicians and 1 imaging physician) according to the Diagnostic Criteria for Occupational pneumoconiosis (GBZ 70-2015) in the National Occupational Health Standards promulgated by the People’s Republic of China in 2015. In refer to the 2021 ERS/ATS Guidelines for Interpretation of Routine Pulmonary Function Tests [7], FEV1/FVC < 70% is defined as obstructive pulmonary ventilation dysfunction, and FVC/Pre < 80% is defined as restrictive pulmonary ventilation dysfunction.

The exposure level reflects the cumulative exposure amount (exposure time × concentration). When the concentration of the same job is equal, the exposure level depends on the effective exposure, that is, the length of underground exposure time; High level exposure refers to downhole workers with long effective exposure time, including air drill, excavation and transportation workers, who are exposed to the underground for 6–8 h per day. Low level exposure refers to short effective exposure time, part of the work in the well, part of the work in the well, mainly including: management, safety, maintenance, etc., about 2–4 h per day in the underground exposure.

2.3 SPSS 26.0 software was used for statistical analysis. The measurement data were expressed as mean ± standard deviation, and statistical analysis was performed by T test. The count data were expressed as frequency and percentage, and were statistically analyzed using the χ² test or Fisher precision test. The risk factors of obstructive pulmonary ventilation dysfunction were analyzed by binary logistic regression. *P* < 0.05 was considered statistically significant.

## Results

1. 3.1 The average age of silica dust exposed workers (*n* = 2064) was 43 years old, the oldest age was 63 years old, the youngest age was 19 years old, and the proportion of over 50 years old was 33.7%; The average working age was 16.6 years, 318 women, accounting for 15.4%; 44.1% of those with more than 15 years of service, 39.7% of smoking and 22.5% of high level exposure. (See Table 1)
2. 3.2 Among silica dust exposed workers, the prevalence of obstructive pulmonary ventilation dysfunction was 2.3%, and the prevalence of restrictive pulmonary ventilation dysfunction was 8.1%. The rate of obstructive pulmonary ventilation dysfunction in female group was higher than that in male group (5.3% vs. 1.7%, *p* = 0.00). The prevalence of obstructive pulmonary ventilation function in the high exposure group was higher than that in the low exposure group (8.2% vs. 0.9%, *p* = 0.00). In 2064 cases of silica dust exposed workers, restrictive lung ventilation dysfunction increased with the increase of age, which was 5.9% in the group < 40 years old, 8.0% in the group 40–49 years old and 9.9% in the group 50–65 years old, and the difference was statistically significant (*p* < 0.05). In 41 patients with silicosis and suspected silicosis (2.0%), there was no statistical difference between obstructive pulmonary ventilation dysfunction and whether there was silicosis or suspected silicosis, but there was statistical difference between restrictive pulmonary ventilation dysfunction and whether there was silicosis or suspected silicosis (*P* = 0.03). (See Table 1)

**Table 1:** Demographic characteristics of workers exposed to silica dust and prevalence of obstructive (FEV1/FVC < 70%) and restrictive (FVC/Pre < 80%) pulmonary ventilation dysfunction

Demographic characteristics	Silica dust			
	Sample size	FEV_1_/FVC < 70%		FVC/Pre < 80%
	2064	47(2.3)		168(8.1)
**Age (years)**				
< 40	508(24.6)	10(2.0)		30(5.9)
40–49	861(41.7)	17(2.0)		69(8.0)
50–65	695(33.7)	20(2.9)		69(9.9)
*P*-values		0.43		0.00
**gender**				
Male	1746(84.6)	30(1.7)		142(8.1)
female	318(15.4)	17(5.3)		26(8.1)
*P*-values		0.00		0.98
female				
≤ 5	249(12.1)	3(1.2)	22(8.8)	
6–15	555(26.9)	5(0.9)	52(9.4)	
16–30	440(21.3)	5(1.1)	34(7.7)	
>30	192(9.3)	3(1.6)	25(13.0)	
N=	628(30.4)	31(4.9)	35(5.6)	
*P*-values		0.90	0.21	
**Exposure Level**				
High	465(22.5)	38(8.2)	44(9.5)	
Low	1003(48.6)	9(0.9)	92(9.2)	
N=	596(28.9)	0(0)	32(5.4)	
*P*-values		0.00	0.86	
**Smoking or not**				
Never	523(25.3)	4(0.8)	47(9.0)	
often	819(39.7)	13(1.6)	69(8.4)	
N=	722(35.0)	30(4.2)	52(7.2)	
*P*-values		0.20	0.72	
**Pneumoconiosis or suspected pneumoconiosis**				
Yes	41(2.0)	1(2.4)	7(17.1)	
no	2023(98.0)	46(2.3)	161(8.0)	
*P*-values		0.94	0.03	

3.3.1 Among the workers exposed to silica dust, 8.2% of workers in the high-level exposure group had obstructive pulmonary ventilation dysfunction, 0.9% of workers in the low-level exposure group had obstructive pulmonary ventilation dysfunction, and the difference between the two groups was statistically significant (*p* < 0.05). The obstructive pulmonary ventilation dysfunction accounted for 5.3% of female workers and 1.7% of male workers, and the difference between the two groups was statistically significant (*p* < 0.05).Workers with obstructive pulmonary dysfunction were older and worked longer than workers without obstructive pulmonary dysfunction, but there was no statistical difference (See Table 2).

**Table 2:** Subgroup analysis of general characteristics of workers exposed to silica dust with and without obstructive pulmonary ventilation dysfunction (FEV1/FVC < 70%)

	*n* = 2064	silicious dust FEV_1_/FVC < 70%		*p*-values
		Yes(*n* = 47)	No(*n* = 2017)	
**Age (years)**	44.9 ± 8.1	47.0 ± 8.7	44.9 ± 8.1	0.08
**seniority**	16.7 ± 10.1	18.0 ± 12.1	16.64 ± 10.1	0.59
**gender**				
Male	1746(84.6)	30(1.7)	1716(98.3)	
female	318(15.4)	17(5.3)	301(94.7)	0.00
**Pneumoconiosis or suspected pneumoconiosis**				
yes	41(2.0)	1(2.4)	40(97.6)	
no	2023(98.0)	46(2.3)	1977(97.7)	0.94
**Exposure Level**				
High	465(22.5)	38(8.2)	427(91.8)	
Low	1003(48.6)	9(0.9)	994(99.1)	0.00
N=	596(28.9)	0(0)	596(100.0)	
**Smoking or not**				
Never	523(25.3)	4(0.8)	519(99.2)	
often	819(39.7)	13(1.6)	806(98.4)	0.20
N=	722(35.0)	30(4.2)	692(95.8)	

3.3.2 In all age groups (< 40 years old, 40–49 years old, 50–65 years old), the prevalence of obstructive pulmonary ventilation disorder in the high level exposure group was significantly higher than that in the low level exposure group, and the differences were statistically significant(*p* < 0.05); The prevalence of obstructive pulmonary ventilation disorder in the high level exposure group of smoking group and non-smoking group was significantly higher than that in the low level exposure group, and the differences were statistically significant(*p* < 0.05);(See Table 3).

**Table 3:** Prevalence rate of workers exposed to silicon dust at different levels

Project	Exposure level of silica dust				*p*-values
	High (465)		Low (1003)		
	*n*	FEV_1_/FVC < 70%	*n*	FEV_1_/FVC < 70%	
**Age (years)**					
< 40	99(21.3)	10(10.1)	266(26.5)	0(0)	0.00
40–49	205(44.1)	13(6.3)	405(40.4)	4(1.0)	0.00
50–65	161(34.6)	15(9.3)	332(33.1)	5(1.5)	0.00
*p*-values		0.43		0.15	
**seniority(years)**					
≤ 5	68(14.6)	0(0)	171(17.0)	3(1.8)	0.27
6–15	194(41.7)	4(2.1)	371(37.0)	1(0.3)	0.03
16–30	146(31.4)	3(2.1)	294(29.3)	2(0.7)	0.20
>30	27(5.8)	2(7.4)	165(16.5)	1(0.6)	0.01
N=	30(6.5)	29(96.7)	2	2(100.0)	
*p*-values		0.15		0.29	
**Smoking or not**					
Never	114(24.5)	9(7.9)	346(58.9)	4(1.2)	0.00
often	250(53.8)	4(1.6)	495(41.1)	0(0)	0.00
N=	101(21.7)	25(24.8)	162	5(3.1)	
*p*-values		0.00		0.02	
**Pneumoconiosis or suspected pneumoconiosis**					
yes	30(6.5)	1(3.3)	11(1.1)	0(0)	0.54
no	435(93.5)	37(8.5)	992(99.0)	9(0.9)	0.00
P-values		0.32		0.75	

3.3.3 There was no significant difference in the prevalence of obstructive pulmonary ventilation dysfunction between the smoking and non-smoking groups. However, in the never-smoking group, the prevalence of obstructive pulmonary ventilation dysfunction increased with age, and the difference was statistically significant(*p* < 0.05). In both smoking and never smoking groups, the prevalence of obstructive pulmonary ventilation dysfunction in the high silica dust exposure group was higher than that in the low silica dust exposure group, with statistical significance. (See Table 4)

**Table 4:** Prevalence rate of silicon dust among workers with different smoking levels

Project	Smoking level				*p*-values
	often (819)		Never (523)		
	*n*	FEV_1_/FVC < 70%	*n*	FEV_1_/FVC < 70%	
**Age (years)**					
< 40	237(28.9)	3(1.3)	120(22.9)	0(0)	0.22
40–49	302(36.9)	6(2.0)	250(47.8)	2(0.8)	0.25
50–65	280(34.2)	4(1.4)	153(29.3)	5(3.3)	0.20
*p*-values		0.77		0.04	
**seniority(years)**					
≤ 5	108(13.2)	1(0.9)	58(11.1)	0(0)	0.46
6–15	304(37.1)	2(0.66)	173(33.1)	1(0.6)	0.92
16–30	206(25.2)	3(2.1)	170(32.5)	2(1.2)	0.81
>30	122(14.9)	2(1.5)	59(11.28)	1(1.7)	0.98
N=	79(9.6)	5(6.3)	63(12.0)	0(0)	
*p*-values		0.76		0.72	
**Exposure Level**					
Low	495(60.4)	4(0.8)	346(66.2)	0(0)	0.09
High	250(30.5)	9(3.6)	114(21.8)	4(3.5)	0.97
N=	74(9.0)	0(0)	63(12.0)	0(0)	
*p*-values		0.01		0.00	
**Pneumoconiosis or suspected pneumoconiosis**					
yes	22(2.7)	1(4.5)	11(2.1)	0(0)	0.47
no	797(97.3)	12(1.5)	512(97.9)	4(0.8)	0.24
P-values		0.26		0.77	

3.3.4 Finally, the data of 1200 workers exposed to silica dust with no data loss were incorporated into Logistic regression analysis, with whether obstructive pulmonary ventilation dysfunction was the dependent variable (0 = none, 1 = yes), and age (1 = less than 40 years old, 2 = 40–49 years old, 3 = more than 50 years old), gender (1 = male, 2 = female), length of service (1 = less than or equal to 5 years, 2 = 6–15 years, 3 = 16–30 years, 4 = greater than 30 years), smoking status (0 = non-smoking, 1 = smoking), dust concentration, exposure level (0 = low level, 1 = high level), pneumoconiosis or suspected pneumoconiosis (0 = none, 1 = yes) Covariables were included for univariate Logistic regression analysis. Gender and exposure levels that showed statistical differences in univariate regression analysis were included in multivariate Logistic regression analysis. The results suggested that high exposure level was a risk factor for obstructive pulmonary ventilation dysfunction in silica dust exposed workers (*P* = 0.000). Females were a risk factor for obstructive pulmonary ventilation dysfunction (*P* = 0.000). There was no difference in age, smoking, length of service, or presence or absence of pneumoconiosis or suspected pneumoconiosis. (See Table 5)

**Table 5:** Risk analysis of obstructive pulmonary ventilation dysfunction in workers exposed to silica dust

F	B	S.E	*P* *P*-values
gender	1.963	0.351	0.000
Exposure Level	2.709	0.351	0.000

## Discussion

This study investigated the prevalence of obstructive pulmonary ventilation dysfunction in silica dust exposed workers and analyzed its risk factors, and the results showed that obstructive pulmonary ventilation dysfunction (FEV1/FVC&lt; 70%) prevalence was 2.3% (47/2064). This is slightly higher than the findings of another recent cross-sectional study, which showed a prevalence of obstructive pulmonary ventilation dysfunction of 1.47% (30/2045) among occupational dust exposed workers [8]. In the Chinese adult lung health survey, it was reported that the prevalence of COPD in Chinese adults over 20 years old was 8.6%, and that in those over 40 years old was 13.7% [9]. Although the research methods were not completely comparable, it was found that the prevalence of obstructive pulmonary ventilation dysfunction in dust exposed workers was significantly lower than that in the normal population. On the one hand, the workers with occupational contraindications were excluded from the pre-work physical examination, and the workers with respiratory diseases found in the on-the-job physical examination were transferred from their jobs in time, or resigned, that is, the healthy worker bias effect. On the other hand, dust-induced lung function injury is more dominated by restricted pulmonary ventilation dysfunction (FVC decline) [10], which was also confirmed in our study, and the prevalence rate of restricted pulmonary ventilation dysfunction reached 8.1% among workers exposed to silica dust. Similar results were also found in another study. The prevalence of obstructive pulmonary ventilation dysfunction was 1.47% (30/2045), while that of restrictive pulmonary ventilation dysfunction was 12.81% [8]. The degree and speed of FVC decline were significantly higher than FEV1, resulting in the ratio of FEV1 to FVC tending to be normal. Therefore, the prevalence of obstructive pulmonary ventilation dysfunction is too low.

The working age refers to the working years, not exactly equivalent to the effective exposure time of dust, dust concentration in the same mine, the same workplace is roughly the same, but different positions, different types of work (such as mining and management) effective exposure time is very different, so the working age or dust concentration can not reflect the cumulative exposure amount of dust in a position. Therefore, we analyzed the exposure level, which reflects the product of exposure time and dust exposure concentration. High exposure level mainly refers to underground workers (air drilling, digging, transportation), with long effective exposure time and large accumulated dust exposure, while low exposure level has short effective exposure time (management, safety, maintenance, etc.) and small accumulated dust exposure. The prevalence of obstructive pulmonary ventilation dysfunction in workers with high levels of silica dust exposure was 8.2%, which was significantly higher than 0.9% in the group with low levels of exposure. In subgroup analysis, the prevalence of obstructive pulmonary ventilation dysfunction in the high silica dust exposure group was higher than that in the low silica dust exposure group, and the differences were statistically significant. In different age groups, the prevalence of obstructive pulmonary ventilation dysfunction in the high silica dust exposure group was higher than that in the low silica dust exposure group, and the difference was statistically significant. Finally, regression analysis also suggested that high silica dust exposure was a risk factor for obstructive pulmonary ventilation dysfunction (*P* = 0.00).This is consistent with the conclusion that a large number of previous studies have confirmed that there is a dose-effect relationship between the loss of lung function and dust exposure in both smokers and non-smokers [11]. Therefore, it can be concluded that high exposure to silica dust can lead to obstructive pulmonary ventilation dysfunction.

The relationship between smoking and obstructive pulmonary ventilation dysfunction has been confirmed by many studies and has become a consensus. In this study, the smoking rate of silica dust exposed workers was as high as 61.0% (819/1342), and the incidence of obstructive pulmonary ventilation dysfunction in the never smoking group was lower than that in the regular smoking group by 0.8%(4/523) vs. 1.6% (13/819), but the difference was not statistically significant. Multivariate analysis showed no significant relationship between smoking and obstructive pulmonary ventilation dysfunction. Due to the lack of smoking data, this study did not quantify the smoking situation and analyze it in groups. Smoking is one of the main factors leading to obstructive pulmonary ventilation dysfunction. Therefore, when workers have both smoking and dust exposure, which are two risk factors causing lung function injury, the synergistic effect of smoking and dust exposure in the development of obstructive pulmonary ventilation dysfunction should be considered [12].

Age is often listed as a risk factor for COPD. In this study, workers with obstructive pulmonary dysfunction were older and had longer working years than those without obstructive pulmonary dysfunction, but the difference was not statistically significant. However, in the never-smoking group, the prevalence of obstructive pulmonary ventilation dysfunction increased with age; Regression analysis also suggested that age was a risk factor for obstructive pulmonary ventilation dysfunction. It is not clear whether healthy aging causes individuals to be sensitive to COPD or whether age reflects the sum of cumulative dust exposure.

Previous studies believed that the incidence of COPD in men was higher than that in women. However, with the change of smoking patterns, some studies found that women were more susceptible to the harmful effects of smoking than men [13], in the case of the same amount of smoking, and women had a greater burden of small airway diseases [14, 15]. Studies have also found that long-term exposure to metals and chlorinated solvents can cause obstructive pulmonary ventilation dysfunction, and this effect is strongest in women [16]. These results are consistent with the results of this study, that is, women are the risk factors for obstructive pulmonary ventilation dysfunction, suggesting that female lung function is more susceptible to dust, and women with dust work are more likely to suffer from obstructive pulmonary ventilation dysfunction than men.

Our study has certain advantages, reporting the prevalence of silica-dust exposed workers and the prevalence among different subgroups, exploring the reasons for the low prevalence of obstructive pulmonary ventilation dysfunction and the risk factors for the disease, and providing reference for the development of diagnostic criteria for chronic obstructive pulmonary disease caused by occupational dust exposure.This study has some limitations. Data such as silica dust exposure of workers smoking are missing, and smoking history is not quantified. Due to the epidemic, only the results of obstructive pulmonary ventilation dysfunction were collected, and the bronchodilation test was not completed. In addition, we did not collect dust concentrations of silica dust, so we did not analyze the relationship between silica dust exposure and prevalence. According to the current prevalence rate calculation, our present sample size is insufficient, and it is suggested that a larger sample size cohort study can be conducted in the future.

## Conclusion

Silica dust exposure can cause obstructive pulmonary ventilation dysfunction and lead to chronic obstructive pulmonary disease. High level of exposure is a risk factor for obstructive pulmonary ventilation dysfunction. Women exposed to dust are more prone to obstructive pulmonary ventilation dysfunction than men. Early diagnosis of chronic obstructive pulmonary disease caused by silica dust and timely intervention measures are very important to delay the decline of lung function and protect the health of workers.

## Data Availability

The data that support the findings of this study are not openly available due to reasons of sensitivity and are available from the corresponding author upon reasonable request. Data are located in controlled access data storage at Hunan Prevention and Treatment Institute for Occupational Diseases.

## Declarations

### Data sharing statement

The data that support the findings of this study are not openly available due to reasons of sensitivity and are available from the corresponding author upon reasonable request. Data are located in controlled access data storage at Hunan Prevention and Treatment Institute for Occupational Diseases.

## References

[1] Global Strategy for the. Diagnosis, Management, and Prevention ofChronic Obstructive Pulmonary Diseas (2023 REPORT)[EB/OL]. https://goldcopd.org/[J].

[2] Global regional (2021). National burden of stroke and its risk factors, 1990–2019: a systematic analysis for the global burden of Disease Study 2019[J]. Lancet Neurol.

[3] Adeloye D, Chua S, Lee C (2015). Global and regional estimates of COPD Prevalence: systematic review and meta-analysis[J]. J Glob Health.

[4] Global regional (2016). National incidence, prevalence, and years lived with disability for 310 diseases and injuries, 1990–2015: a systematic analysis for the global burden of Disease Study 2015[J]. Lancet.

[5] Global regional. and national deaths, Prevalence, disability-adjusted life years, and years lived with disability for chronic obstructive pulmonary disease and asthma, 1990–2015: a systematic analysis for the Global Burden of Disease Study 2015[J]. Lancet Respir Med, 2017,5(9):691–706. 10.1016/S2213-2600(17)30293-X PMC5573769 28822787

[6] Blanc PD, Toren K (2007). Occupation in chronic obstructive pulmonary disease and chronic bronchitis: an update[J]. Int J Tuberc Lung Dis.

[7] Stanojevic S, Kaminsky DA, Miller MR (2022). ERS/ATS technical standard on interpretive strategies for routine lung funcion tests. Eur Respir J.

[8] He W, Jin N, Deng H. Workers’ Occupational Dust Exposure and Pulmonary Function Assessment: Cross-Sectional Study in China. Int J Environ Res Public Health 2022 Sep 4;19(17):11065. 10.3390/ijerph191711065 PMC9518133 36078779

[9] Wang C, Xu J, Yang L, China Pulmonary Health Study Group (2018). Prevalence and risk factors of chronic obstructive pulmonary disease in China (the China Pulmonary Health [CPH] study): a national cross-sectional study. Lancet.

[10] Paul C. Occupational lung disorders [M]. Fourth Edition[J]. New York.CRCPress.2017.415-432.

[11] Hnizdo E, Vallyathan V. Chronic obstructive pulmonary disease due to occupational exposure to silica dust: a review of epidemiological and pathological evidence. Occup Environ Med. 2003 Apr;60(4):237–43. 10.1136/oem.60.4.237 PMC1740506 12660371

[12] Peng Y, Li X, Cai S, Chen Y, Dai W, Liu W, Zhou (2018). Zijing;Duan, Jiaxi;Chen, Ping(*), prevalence and characteristics of COPD amongpneumoconiosis patients at an occupational disease prevention institute: a cross-sectional study. BMC Pulm Med.

[13] Amaral AFS (2017). Strachan DP,Burney PGJ,Jarvis DL.Female smokers are at Greater risk of airflow obstruction Than Male Smokers.UK Biobank.Am. J Respir Crit Care Med.

[14] Martinez FJ, Curtis JL, Sciurba F (2007). Sex differences in severe pulmonary emphysema. Am J Respir Crit Care Med.

[15] Tam A, Churg A,Wright JL (2016). Sex differences in Airway Remodeling in a mouse model of Chronic Obstructive Pulmonary Disease. Am J Respir Crit Care Med.

[16] Alif SM, Dharmage SC, Benke G, et al. Occupational exposures to solvents and metals are associated with fixed airflow obstruction. Scand J Work Environ Health. 2017 Nov;43(1):595–603. 10.5271/sjweh.3662 28782791

